# The Reproductive Capacities of the Calanoid Copepods *Parvocalanus crassirostis* and *Acartia pacifica* under Different pH and Temperature Conditions

**DOI:** 10.3390/ani13132160

**Published:** 2023-06-30

**Authors:** Montaha Behbehani, Saif Uddin, Nazima Habibi, Hanan A. Al-Sarawi, Yousef Al-enezi

**Affiliations:** 1 Environment and Life Sciences Research Center, Kuwait Institute for Scientific Research, Safat 13109, Kuwait; 2 Environment Public Authority, Al Shuwaikh 13104, Kuwait

**Keywords:** acidification, global warming, experiments, carbon dioxide, reproductive capacities, eggs

## Abstract

**Simple Summary:**

Climate change has negatively affected marine organisms. This experimental study presents data from a multigenerational experiment on the egg production of two commonly occurring calanoid copepods, *Parvocalanus crassirostis* and *Acartia pacifica*, under different pH and temperature conditions. The results suggest that pH and temperature conditions influence the number of eggs produced by healthy copepod pairs. However, when the pH changes were gradually carried out over 14 generations, there was no significant difference in the egg production rate at ambient and lower pH. This suggests that copepod populations might be resilient to future ocean scenarios of lower pH and higher temperature.

**Abstract:**

The increasing atmospheric CO_2_ concentrations and warming of marine waters have encouraged experiments on multi-stressor interactions in marine organisms. We conducted a multigenerational experiment to assess reproductive capacities regarding egg production in calanoid copepods *Parvocalanus crassirostis* and *Acartia pacifica* under different pH and temperature conditions. The experimental set-up allowed assessing the tandem effect of warming and acidification on the number of eggs produced by healthy copepod pairs under two pH conditions of 8.20 and 7.50 (hard selection) as well as with a gradual reduction of 0.05 pH units at each generation (soft selection). The results are quite interesting, with very diverse performance across temperatures. The number of eggs produced under hard selection was higher at pH 8.20 compared to pH 7.50 for both species, with the maximum number of eggs produced at 24–28 °C, whereas under soft selection, there was no significant difference in the egg production rate at 24–28 °C across generations and there was an improvement in the number of eggs produced at 8–16 °C. The results provide evidence that in a future ocean scenario of lower pH and higher temperature, the two species, and possibly the copepod population at large, might not decrease. Copepod populations might be resilient, and the transcriptomic evidence of adaptation to increased temperature and lower pH is a ray of hope. We believe further studies are needed to provide more robust datasets to underpin the hypothesis of adaptation to climate change.

## 1. Introduction

During the last few decades, the Persian Gulf has witnessed ocean warming (OW) and ocean acidification (OA) that perturbs the seawater chemistry and possibly stresses the marine ecosystem [1,2,3,4]. Understanding the ecological effect of OW and OA is a priority area for marine research, particularly when fisheries resources are a critical renewable food source for the Gulf region. Most studies conducted so far have highlighted dwindling fish stocks and reduced fish landings in the region that are primarily attributed to improper fisheries management. There is a need to consider other aspects, including OW and OA, which have been widely reported to harm fisheries [5]. Zooplanktons are an important link between primary producers and fish. Copepods are primary consumers in the ocean and the most numerous metazoans, supporting the marine food web and acting as a biological pump of carbon [6]. Any effect on the abundance and diversity of copepods due to a drop in pH and warming of the seawater is likely to have severe implications for the functioning of the marine ecosystem. Studies show the lethality of increasing pCO_2_ levels on both surface and deep-sea copepod species [7]; however, deep-sea copepods demonstrate tolerance to higher *p*CO_2_. It can be hypothesized that this higher tolerance is probably an adaptation to the high-*p*CO_2_ environment where they live. It has been reported that OW causes oxidative stress and a reduction in the reproductive capacity of *Acartia bifilosa* [8]. However, the magnitude of these responses varies between species and spatially in different regions [7,9,10]. The Gulf ecosystem is somewhat unique for its temperature, salinity, nutrient loadings, and pH and has shown resilience to these changes (i.e., corals are surviving at a higher temperature in turbid water; breeding grounds for various fin and shellfish are in areas reported to be exposed to potentially stressing conditions) [11,12,13,14]. No experimental studies have been conducted in the region to quantify the impact of OW and OA on copepods, which are a vital link in the marine food web.

The water of the northern Persian Gulf shows a drop in pH primarily attributed to the oceanic uptake of CO_2_ and dry climate and reduced freshwater input to an increase in sea surface temperature [13,14,15]. This study is designed to understand the effect of two significant phenomena, OA and OW, on the reproductive capacity of *Parvocalanus crassirostis* and *Acartia pacifica*, using both abrupt change and slow long-term transgenerational experimental designs and a wide range of treatments to identify if these parameters can affect the abundance of copepods within the marine food web.

## 2. Material and Methods

The copepod samples were collected using a 2 m long, 0.6 m diameter, 50 µm mesh size plankton net. The samples were collected between February and April 2019 in the northern Persian Gulf, covering a large number of spots by making vertical plankton tows from bottom to surface. The depth varied between 10 to 30 m from the surface. At each station, the sea surface temperature, pH, and salinity were also recorded. The temperature was between 22.3 and 29.5 °C, the pH was between 8.09 and 8.18, and the salinity was between 42.8 and 44.3 ppt. All the samples obtained from a single location were placed in filtered seawater and immediately transported to the laboratory for sample identification and segregation to create a monoculture. The sample was emptied into a Bogorov counting chamber and examined under a stereomicroscope. Fine needles and pipettes were used to isolate the identified species for preparing a monoculture. Samples collected on a single cruise day from different areas within the territorial water were segregated at the lowest taxonomic level by specialists in copepod identification and following the characteristics of the species in an identification guide [16]. All the samples of single species from different areas were combined into a single beaker with filtered seawater. Once the monoculture was segregated, they were transferred into 5 l beakers. Within the beakers, the copepods were housed in a cylindrical enclosure with a mesh base. The first screen was 90 µm in size, the second screen was 75 µm, and the third was 20 µm. With this sequential sieving, the adults, nauplii, eggs, and fecal matter were efficiently separated. The fraction collected on the 90 µm screen was used in the experiment, while copepods in the 75 and 20 µ sieves were transferred into a smaller Petri dish and concentrated to the center with a slow rotating movement [17]. Through this process, the eggs were separated from nauplii I-IV and detritus for the multi-generational experiment.

**Figure 1:**
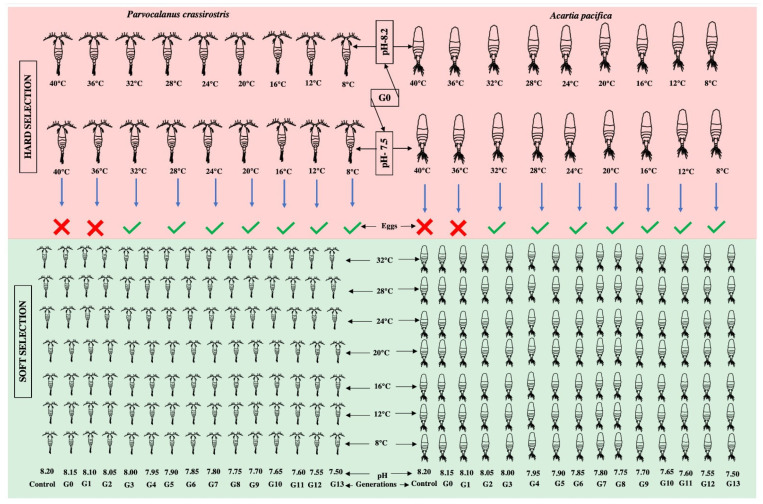
The graphical representation of the experimental design.

The experiment was designed to compare the different rates of change (over one generation subjected to two extreme pHs and different temperatures, which we refer to as “hard selection”, and a reduction in pH by 0.05 pH units over each generation, referred to as “soft selection” (Figure 1). A wide range of temperatures were covered to resolve the performance curves for different *p*CO_2_ scenarios represented as the reproductive capability of adults (in terms of the number of eggs produced) under a combination of varying pHs and temperatures.

In the case of hard selection, three *p*CO_2_ scenarios were compared: (i) A constant pH of 8.20 (control); (ii) an abrupt exposure to pH 7.50, out of the present range of pH variability, and expected by 2100; and (iii) a step-wise pH reduction of 0.05 pH units per generation over 13 generations (“soft selection”). Nine temperatures (40.0, 36.0, 32.0, 28.0, 24.0, 20.0, 16.0, 12.0, and 8.0 °C) covering the current and future range of natural variability at the sampling site were tested. The temperature and pH were regulated using a computer-controlled IKS Aquastar system (IKS ComputerSysteme GmbH, Karlsbad, Germany).

Healthy adult females and males of *Parvocalanus crassirostis* and *Acartia pacifica* were placed on six-well cell culture plates adjusted to 8.20 and 7.50 pH for hard selection and for G0, with the pH being decreased by 0.05 for each subsequent generation, but 8.20 being kept as the control; thus a total of 3 pH conditions in each generation. A total of 18 pairs of *Parvocalanus crassirostis* and *Acartia pacifica* were used for each pH and temperature treatment to undertake survival and egg production experiments, i.e., 3 culture plates with 6 wells, with a pair in each well for each condition. The cell plates were covered with parafilm to minimize CO_2_ exchange, and the temperature was regulated by keeping these cell plates in thermoregulated baths. The pH and temperature were measured daily and adjusted if required during the experiment. The feeding was carried out using a pipette. After 24 h, the produced eggs were isolated under a dissecting microscope to minimize cannibalism by adult copepods and transferred into a new six-well cell culture plate with the same pH and temperature conditions. There was no mortality of adult copepods observed over the 24 h period and the number of produced eggs was counted. The experiment was continued for thirteen generations (G1–G13) following a similar protocol.

### Statistical Analysis

Two-way ANOVA (analysis of variance) was conducted to compare the means of individual experiments [18]. The fixed effect model, without any repetitions and with interaction, was employed. The means were compared at a confidence interval of 95% (ɑ-0.05). Prior to this, the samples were checked for outliers through Tukey’s fence test (k = 1–5). The outliers were in the expected range of 4.97%. The test power for both the factors (pH and temperature) was strong. A residual test was employed to check the normality of the data using the Shapiro–Wilk test (q = 0.05), suggesting a normal distribution of residuals. The test design was balanced at 98.8%. These tests were conducted in the graphical user interface available online at www.statisticskingdom.com. The R codes for the tests are available on the website. A Wilcoxon signed-rank test was used to observe the standardized effect of pH 8.20 and 7.50 on the population of *P. crassirostris* and *A. pacifica*. The Kruskal–Wallis H-test was employed to calculate the impact on the size, test power, outliers, and R syntax. This was followed by Dunn’s/Mann–Whitney’s post hoc test using a Bonferroni-corrected alpha [19] to compare the means of pH and temperature over thirteen generations of both the copepod species. The output of the statistical tests was imported into Numbers 12.1 (Macintosh HD, Apple Inc., Cupertino, CA, USA) and plotted as line charts or scatter plots.

## 3. Results

### 3.1. Hard Selection of Temperature

**Figure 2:**
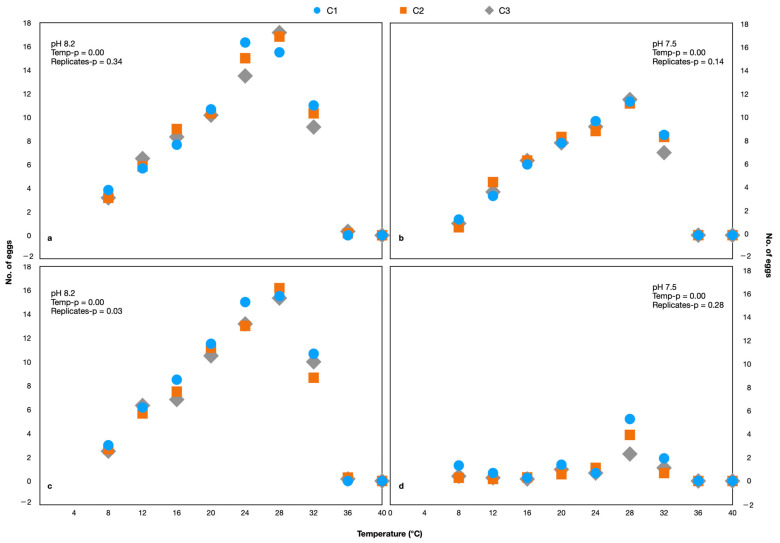
The number of eggs produced by *Parvocalanus crassirostris* (**a**,**b**) and *Acartia pacifica* (**c**,**d**). The C1, C2, and C3 are replicates.

**Figure 3:**
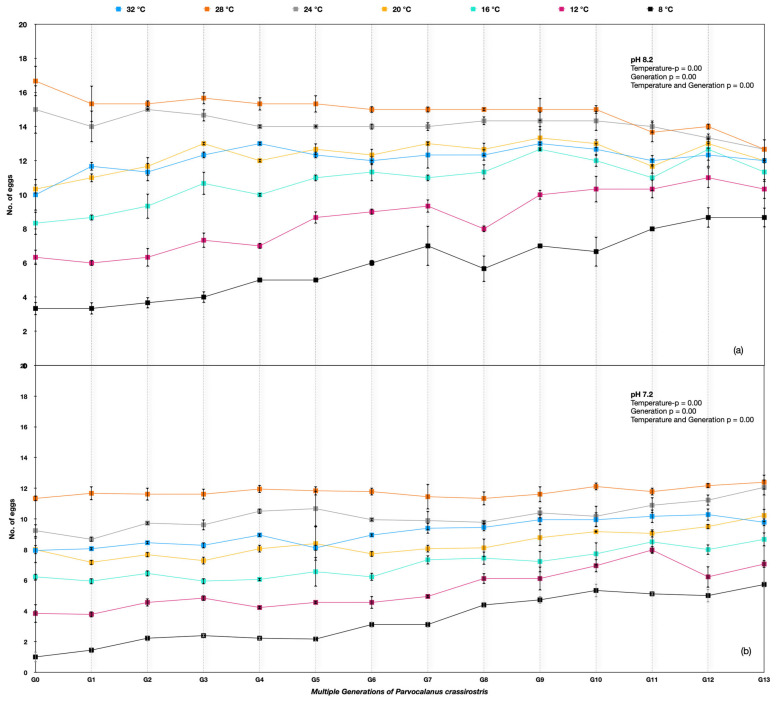
*Parvocalanus crassirostris* G0 to G13 generations at the hard selection of pH (**a**) 8.20 and (**b**) 7.50.

During each generation, the egg production rate was determined to assess the reproductive capability. There were no eggs produced at 40 °C and only one egg was produced at 36 °C and 8.20 pH, while there were no eggs produced at these temperatures at 7.50 pH (Figure 2). Since there were no eggs produced or which survived at 36 and 40 °C, this range was excluded from the experiment of G1 onwards. A comparison of the number of eggs produced by each generation under different pH and temperatures is shown in Figure 3 (Supplementary Table S1).

### 3.2. Parvocalanus Crassirostis

The egg production rate differed significantly among different temperature treatments across the generations for *Parvocalanus crassirostis*. A two-way ANOVA was used, and the *p*-value was <0.005 between temperatures, indicating significant variations, whereas the *p*-value > 0.005 between replicates showed nonsignificant variation. Considerable variation between generations G0 and G13 was observed under the ambient pH of 8.20 and at the lower pH of 7.50. The egg production rates improved under 8 °C, 12 °C, 16 °C, and 20 °C temperatures over the generations at the lower pH of 7.5. The best performance in the number of eggs produced was at 24 °C and 28 °C, with no change in egg production rates at 8.20 pH and a slight improvement across the generations with soft selection (Figure 3).

**Figure 4:**
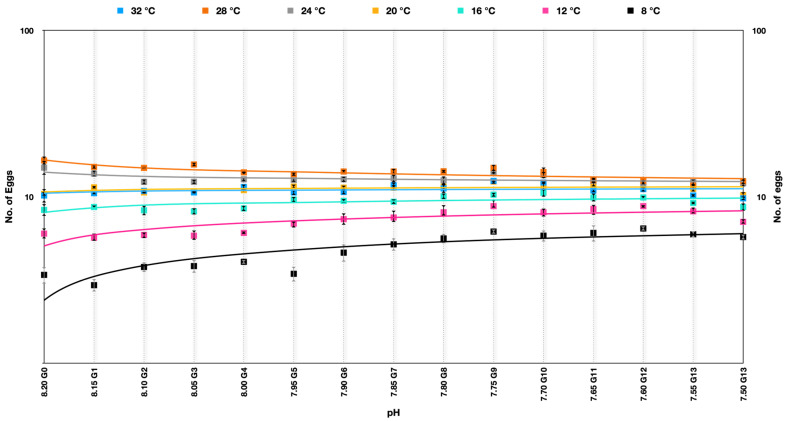
Number of eggs produced by *Parvocalanus crassirostris* at a slow selection of pH. The *x*-axis shows the drop in pH, whereas the log_10_ counts of eggs are plotted on the *y*-axis.

The egg production rate in *Parvocalanus crassirostris* under soft selection depicted a very interesting outcome quite different from hard selection across the experimental temperatures. The best performance was at temperatures of 24–28 °C across the generations and was quite consistent across the pHs over generations. At temperatures of 32 °C, 20 °C, and 12 °C, the egg production rate was more or less consistent over the generations, whereas there was a significant improvement in the egg production rate at 8 °C and 12 °C (Figure 4) (Supplementary Table S1).

The Kruskal–Wallis H-test indicated that there was a significant difference in the dependent variable (temperature in this case) between the different groups (χ^2^(6) = 96.01, *p* < 0.001), with a mean rank score of 58.53 for 32, 94.03 for 28, 85.7 for 24, 62.87 for 20, 37.83 for 16, 22.7 for 12, and 9.33 for 8 (Table 1). This was followed by the post-hoc Dunn’s test using a Bonferroni-corrected alpha of 0.0024, indicating that the mean ranks of the following pairs were significantly different: 32-28, 32-12, 32-08, 28-16, 28-12, 28-08, 24-16, 24-12, 24-08, 20-12, and 20-08.

The Kruskal–Wallis H-test indicated that there was a nonsignificant difference (Table 2) in the dependent variable (gradient of pH in this case) between the different groups under soft selection (χ^2^(14) = 3.13, *p* = 0.999), with a mean rank score of 49.93 for 8.20, 50.86 for 8.15, 48 for 8.10, 48.07 for 8.05, 52.14 for 8.00, 52.07 for 7.95, 53.36 for 7.90, 56.14 for 7.85, 56.21 for 7.80, 65.71 for 7.75, 60.29 for 7.70, 55.79 for 7.65, 54.5 for 7.60, 47.71 for 7.505, and 44.21 for 7.50 (Table 2). This nonsignificant difference shows that these groups with different pH were comparable.

### 3.3. Acartia Pacifica

**Figure 5:**
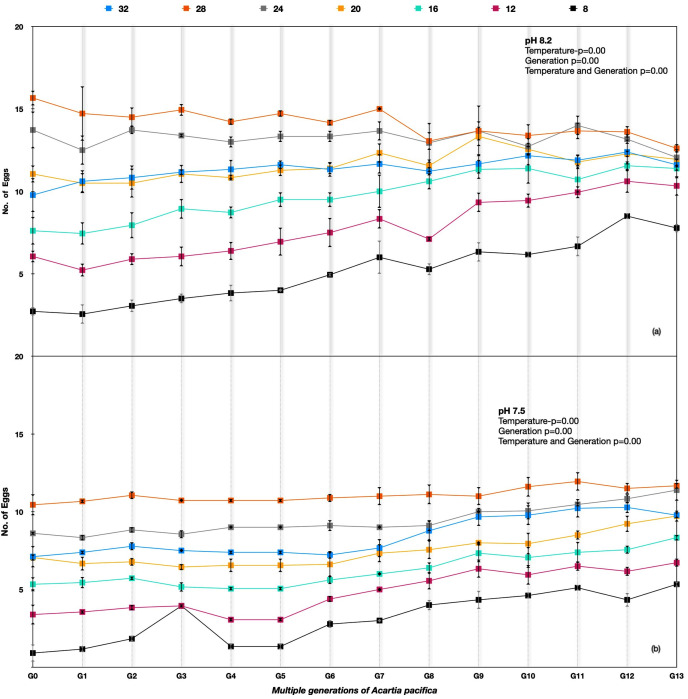
*Acartia pacifica* G0 to G13 generations at the hard selection of pH (**a**) 8.20 and (**b**) 7.50.

The performance in terms of the number of eggs per healthy female for *Acartia pacifica* was highest for 24–28 °C at all the pH treatments. The egg production rates differed significantly among different temperature treatments across the generations for *A. pacifica* (Table 3). Considerable variation between generations G0 and G13 was observed under an ambient pH of 8.20; the egg production rates improved under 8 °C, 12 °C, and 16 °C temperatures over the generations. However, the best performance in the number of eggs produced at 8.20 pH was at 24 °C and 28 °C, with a reduction in the number of egg production rates at 8.20 pH in the following generations (Figure 5a), whereas at the lower pH of 7.50, there was an improvement in the egg production rates across the generations at all temperatures (Figure 5b).

The two-way ANOVA showed the *p*-value was <0.005 between multiple generations of *A. pacifica* females at different temperatures and pH, indicating significant variation.

**Figure 6:**
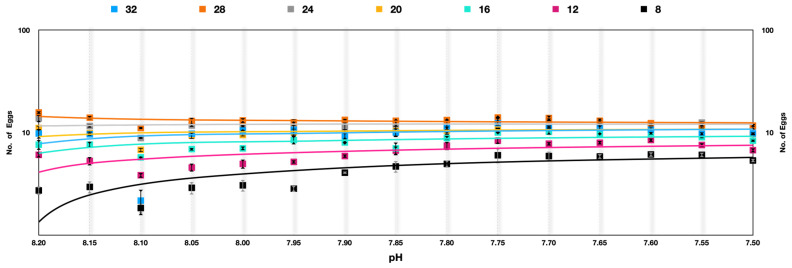
*Acartia pacifica* slow selection of pH, log scale with a logarithmic trendline. Each data point signifies one generation.

The egg production rate among *A. pacifica* under soft selection depicted a very interesting outcome that was quite different from hard selection across the experimental temperatures. The best performance was at temperatures 28 °C > 24 °C > 20 °C > 32 °C > 16 °C > 12 °C > 8 °C across the generations. The difference in egg production rate was reduced in progressive generations, indicating adaptation to pH differences. At temperatures of 28 °C and 24 °C, the egg production rate was more or less similar in later generations. A significant improvement in egg production rate across the generations was observed at 8 °C (Figure 6).

The Kruskal–Wallis H-test indicated that there was a significant difference in the dependent variable between the different groups (χ^2^(6) = 80.74, *p* < 0.001), with a mean rank score of 61.72 for 32, 96.28 for 28, 85.94 for 24, 64.97 for 20, 44.59 for 16, 28.44 for 12, and 13.56 for 8. The post hoc Dunn’s test using a Bonferroni-corrected alpha of 0.0024 indicated that the mean ranks of the following pairs were significantly different: 32–08, 28–16, 28–12, 28–08, 24–16, 24–12, 24–08, 20–16, and 20–08 (Table 3).

The Kruskal–Wallis H-test indicated that there was a nonsignificant difference in the dependent variable between the different groups in the case of soft selection (χ2(14) = 12.36, *p* = 0.577), with a mean rank score of 55.79 for pH 8.20, 48.57 for pH 8.15, 24.07 for pH 8.1, 45 for pH 8.05, 47.79 for pH 8.0, 49.21 for pH 7.95, 48.93 for pH 7.9, 52.21 for pH 7.85, 56.86 for pH 7.8, 67.93 for pH 7.75, 66.79 for pH 7.7, 64 for pH 7.65, 61.36 for pH 7.6, 57.21 for pH 7.505, and 49.29 for pH 7.50 (Table 4). The nonsignificant difference, in this case, represents similarities between the groups that can be interpreted as adaptation.

## 4. Discussion

The egg production rate of *Parvocalanus crassirostis* and *Acartia pacifica* was significantly lower at lower pH, suggesting an increase in *p*CO_2_ concentrations is likely to affect copepods negatively, especially under the hard selection. The result indicates that a sudden pH change is expected to significantly affect the population of calanoid copepods, which are secondary producers in marine ecosystems. Studies have shown that exposure to low-pH seawater can lead to reduced egg production and delayed copepod growth over multiple generations [9,20,21]. *Acartia tonsa,* a calanoid copepod, showed delayed growth at 7.8 pH. Additionally, different harpacticoid copepod species showed varying tolerance levels when exposed to acidified deep-sea sites [22,23].

Despite the increasing number of studies on the effects of ocean acidification on copepods, the amount of information available on their responses is still insufficient, and the effects are not clearly defined. Therefore, further research is needed to determine the full extent of the impacts of ocean acidification on copepods and other marine organisms. This research can help inform effective management strategies to mitigate the effects of ocean acidification on marine ecosystems.

This study showed a significant difference in the egg production rates of both *Parvocalanus crassirostis* and *Acartia pacifica* exposed to different temperatures and pH. The results provide evidence that the best performance in terms of egg production for both species across the range of pHs was at 24–28 °C. The study further underpins the difference in responses between the hard and soft selection. The egg production rates in both *Parvocalanus crassirostis* and *Acartia pacifica* showed improvement over the generations across the temperatures and pHs. These copepods are abundant in the coastal areas of the Gulf and elsewhere, and any change in their abundance could affect the food chain. The finding is consistent with previous studies, where the early life stages were shown to be more susceptible to stress. Likewise, *Amphiascus tenuiremis* nauplii were 28 times more sensitive to contaminant exposure in terms of survival rate than adult individuals [24,25]. The early life stages of copepods have been shown to be more sensitive to CO_2_ storage in deep-sea sites [26,27,28], as well as in other invertebrates [20,29,30,31].

This study becomes more relevant since copepods are a crucial link in the marine food web between primary producers and organisms at higher trophic levels. This study further strengthens the fact that the effects of long-term multi-generation experiments differ from many short-term exposures (single exposure/generation). Experiments using adults might sometimes underestimate the detrimental effects of OA-OW.

We found that the later generations of both the copepods across the temperatures were more resilient to lower pH (increased CO_2_) than the G0. The pH of the seawater from which the copepods were caught was between 8.09 and 8.18, whereas under both hard and soft selection, the endpoint was 7.50. We believe that this is an adaptation, and there is transcriptomic evidence of the upregulation/downregulation of genes that are sensitive to temperature and pH [32]. These results are similar to other studies investigating *T. japonicus*, which showed strong resistance and tolerance at very high *p*CO_2_ concentrations [33,34,35]. Similar to our observations, many studies on harpacticoid copepods have reported differences between individuals collected at sea and cultivated in the laboratory for several generations [36,37,38]. However, these may have developed physiological differences to wild-living individuals. The results of the current study support this conclusion.

## 5. Conclusions

This study underpins the hypothesis that organisms can adapt if the changes in pH and temperature are gradual. The results provide evidence that the best performance in terms of egg production for both *Parvocalanus crassirostis* and *Acartia pacifica* across a range of pHs is at 24–28 °C. It brings forth a vital discussion: Will the abundance of the copepods during a specific season also affect the food availability for other organisms that prey on them? This seasonality in abundance can also have a compounding effect at higher trophic levels, as some of the fish larvae feed on these copepods; the seasonal difference can have serious ramifications on their population.

On the other hand, the effect of pH under soft selection when the pH is lowered slowly over generations was very subtle. There was no significant effect on the number of eggs produced at 8.2 and 7.5 pH after 14 generations. The number of eggs produced at 8.2 pH were consistently higher than 7.5 pH across different temperatures. The lower number of eggs produced at 7.5 pH under hard selection was expected as the ambient pH during collection was 8.09–8.18. These organisms were further acclimatized to 8.2 pH in the laboratory, so a sudden drop of 0.7 pH units was a substantial stress for them. There was absolute mortality at 36 and 40 °C, suggesting this is the inflection point beyond which these copepods will not survive.

The observations are significant because they provide evidence of adaptation when a change in pH is lower. Also, this is true of temperature; the performance across extreme temperatures also improved over generations under soft selection.

## Figures and Tables

**Table 1:** Multiple comparisons of means at varying temperatures through Kruskal–Wallis H-test in *P. crassirostris*.

Group	28	24	20	16	12	8
32	**−35.50**	−27.17	−4.33	20.7	**35.83**	**49.20**
28	0.00	8.33	31.17	**56.2**	**71.33**	**84.70**
24	8.33	0.00	22.83	**47.87**	**63.00**	**76.37**
20	31.17	22.83	0.00	25.03	**40.17**	**53.53**
16	**56.20**	**47.87**	25.03	0.00	15.13	28.50
12	**71.33**	**63.00**	**40.17**	15.13	0.00	13.37

Values in bold indicate a significant difference in means.

**Table 2:** Multiple comparisons of means at varying pH through Kruskal–Wallis H-test in *P. crassirostris*.

Group	8.15	8.1	8.05	8	7.95	7.9	7.85	7.8	7.75	7.7	7.65	7.6	7.505	7.50
8.20	−0.93	1.93	1.86	−2.21	−2.14	−3.43	−6.21	−6.29	−15.79	−10.36	−5.86	−4.57	2.21	5.71
8.15	0	2.86	2.79	−1.29	−1.21	−2.50	−5.29	−5.36	−14.86	−9.43	−4.93	−3.64	3.14	6.64
8.10	2.86	0	−0.07	−4.14	−4.07	−5.36	−8.14	−8.20	−17.71	−12.29	−7.79	−6.5	0.29	3.79
8.05	2.79	−0.07	0	−4.07	−4.00	−5.29	−8.07	−8.14	−17.64	−12.21	−7.71	−6.43	0.36	3.86
8.00	−1.29	−4.14	−4.07	0	0.07	−1.21	−4.00	−4.07	−13.57	−8.14	−3.64	−2.36	4.43	7.93
7.95	−1.21	−4.07	−4.00	0.07	0	−1.29	−4.07	−4.14	−13.64	−8.20	−3.71	−2.43	4.36	7.86
7.90	−2.5	−5.36	−5.29	−1.21	−1.29	0	−2.79	−2.86	−12.36	−6.93	−2.43	−1.14	5.64	9.14
7.85	−5.29	−8.14	−8.07	−4.00	−4.07	−2.79	0	−0.07	−9.57	−4.14	0.36	1.64	8.43	11.93
7.80	−5.36	−8.20	−8.14	−4.07	−4.14	−2.86	−0.07	0	−9.50	−4.07	0.43	1.71	8.50	12.00
7.75	−14.86	−17.71	−17.64	−13.57	−13.64	−12.36	−9.57	−9.5	0	5.43	9.93	11.21	18.00	21.50
7.70	−9.43	−12.29	−12.21	−8.14	−8.20	−6.93	−4.14	−4.07	5.43	0	4.50	5.79	12.57	16.07
7.65	−4.93	−7.79	−7.71	−3.64	−3.71	−2.43	0.36	0.43	9.93	4.50	0	1.29	8.07	11.57
7.60	−3.64	−6.5	−6.43	−2.36	−2.43	−1.14	1.64	1.71	11.21	5.79	1.29	0	6.79	10.29
7.55	3.14	0.29	0.36	4.43	4.36	5.64	8.43	8.50	18.00	12.57	8.07	6.79	0	3.50

All the values have nonsignificant differences in means.

**Table 3:** Comparisons of means at varying temperatures through Kruskal–Wallis H-test in *A. pacifica*.

Group	28	24	20	16	12	8
32	−34.56	−24.22	−3.25	17.13	33.28	48.16
28	0	10.34	31.31	51.69	67.84	82.72
24	10.34	0	20.97	41.34	57.50	72.38
20	31.31	20.97	0	20.38	36.53	51.41
16	51.69	41.34	20.38	0	16.16	31.03
12	67.84	57.50	36.53	16.16	0	14.88

Values in bold indicate a significant difference in means.

**Table 4:** Comparisons of means at varying pH through Kruskal–Wallis H-test in *A. pacifica*.

Group	8.15	8.10	8.05	8.00	7.95	7.90	7.85	7.80	7.75	7.70	7.65	7.60	7.55	7.50
8.20	7.21	31.71	10.79	8.00	6.57	6.86	3.57	−1.07	−12.14	−11.00	−8.20	−5.57	−1.43	6.50
8.15	0	24.50	3.57	0.79	−0.64	−0.36	−3.64	−8.20	−19.36	−18.20	−15.43	−12.79	−8.64	−0.71
8.10	24.50	0	−20.93	−23.71	−25.14	−24.86	−28.14	−32.79	−43.86	−42.71	−39.93	−37.29	−33.14	−25.21
8.05	3.57	−20.93	0	−2.79	−4.21	−3.93	−7.21	−11.86	−22.93	−21.79	−19.00	−16.36	−12.21	−4.29
8.00	0.79	−23.71	−2.79	0	−1.43	−1.14	−4.43	−9.07	−20.14	−19.00	−16.21	−13.57	−9.43	−1.50
7.95	−0.64	−25.14	−4.21	−1.43	0	0.29	−3.00	−7.64	−18.71	−17.50	−14.79	−12.14	−8.00	−0.07
7.90	−0.36	−24.86	−3.93	−1.14	0.29	0	−3.29	−7.93	−19.00	−17.86	−15.07	−12.43	−8.20	−0.36
7.85	−3.64	−28.14	−7.21	−4.43	−3.00	−3.29	0	−4.64	−15.71	−14.57	−11.79	−9.14	−5.00	2.93
7.80	−8.20	−32.79	−11.86	−9.07	−7.64	−7.93	−4.64	0	−11.07	−9.93	−7.14	−4.50	−0.36	7.50
7.75	−19.36	−43.86	−22.93	−20.14	−18.71	−19.00	−15.71	−11.07	0	1.14	3.93	6.57	10.71	18.64
7.70	−18.20	−42.71	−21.79	−19.00	−17.50	−17.86	−14.57	−9.93	1.14	0	2.79	5.43	9.57	17.50
7.65	−15.43	−39.93	−19.00	−16.21	−14.79	−15.07	−11.79	−7.14	3.93	2.79	0	2.64	6.79	14.71
7.60	−12.79	−37.29	−16.36	−13.57	−12.14	−12.43	−9.14	−4.50	6.57	5.43	2.64	0	4.14	12.07
7.55	−8.64	−33.14	−12.21	−9.43	−8.00	−8.20	−5.00	−0.36	10.71	9.57	6.79	4.14	0	7.93

All the values have nonsignificant differences in means.

## Data Availability

The data is available in a supplementary file and additionally in Final Report EM092C, Kuwait Institute for Scientific Research which can be made available on request.

## Supplementary Materials

The following supporting information can be downloaded at: https://www.mdpi.com/article/10.3390/ani13132160/s1, Table S1: Number of eggs produced and replicates under hard and Soft Selections.
